# Oocyte-specific knockout of eIF2B subunits causes apoptosis of mouse oocytes within the early growing follicles via DNA damage and mitochondrial dysfunction

**DOI:** 10.1038/s41419-026-09092-3

**Published:** 2026-07-10

**Authors:** Weiyong Wang, Huiyu Liu, Biao Li, Shuang Liu, Luchun Zhang, Ying Wei, Hongwei Wei, Xiaodan Zhang, Xiaoqiong Hao, Meijia Zhang

**Affiliations:** 1 The Innovation Centre of Ministry of Education for Development and Diseases, School of Medicine, South China University of Technology, Guangzhou, China; 2 Department of Physiology, Baotou Medical College, Baotou, China

**Keywords:** Oogenesis, Apoptosis

## Abstract

Mutations in several translation initiation factors are closely associated with premature ovarian insufficiency (POI). In this study, we demonstrated that the conditional knockout of eukaryotic translation initiation factor 2B (eIF2B) subunits *Eif2b2, Eif2b3, Eif2b4* or *Eif2b5*, but not *Eif2b1*, in mouse oocytes of primordial follicles caused oocyte apoptosis within the early growing follicles. Integrative Ribo-seq and RNA-seq analysis revealed the upregulation of integrated stress response- and DNA damage-related genes, and the downregulation of oocyte development-related genes in *Eif2b4*-cKO oocytes. Then, further research indicated that the depletion of *Eif2b4* in oocytes resulted in mitochondrial dysfunctions characterized by decreased mitochondrial membrane potential, mtDNA copy numbers, and ATP content, along with excessive accumulation of reactive oxygen species (ROS). At the same time, the depletion of *Eif2b4* in oocytes resulted in the increased levels of DNA damage response (DDR) proteins (NBS1, p-ATM, p-CHK2, and p53) and proapoptotic protein BAX, as well as the decreased levels of DNA damage repair protein Polβ and anti-apoptotic protein BCL-xL. Collectively, these findings indicate that the depletion of eIF2B subunits in mouse oocytes leads to oocyte apoptosis within the early growing follicles by DNA damage and mitochondrial dysfunction. This study provides new insights into the pathogenesis and genetic diagnosis of POI.

## Introduction

The ovarian follicle is an indispensable structure to generate mature oocytes and synthesize steroid hormones in mammals [1]. During the perinatal period, the nonrenewable primordial follicles constitute the ovarian reserves, thereby determining reproductive lifespan [2]. Overactivation or failed activation of primordial follicles drives the premature exhaustion of the follicle reserve, leading to premature ovarian insufficiency (POI) [3, 4]. In every follicular wave, only a minority of primordial follicles are recruited and activated. In contrast, the others remain in a state of low metabolic, transcriptional, and translational activity to maintain steady ovarian reserve [2, 5]. Activated oocytes undergo a rapid growth phase characterized by a massive increase in oocyte volume, organelle biogenesis, mRNA, and protein synthesis [6]. During oocyte growth, rapid mitochondrial biogenesis, driven by fission and mitochondrial DNA (mtDNA) replication, provides adenosine triphosphate (ATP) to support protein translation and gene transcription [7, 8]. Simultaneously, high-efficiency synthesis of oocyte-secreted factors such as bone morphogenetic protein 15 (BMP15) and growth differentiation factor 9 (GDF9) enhances proliferation, differentiation, and metabolism of granulosa cells, which in turn support oocyte development and maturation by transporting pyruvate, amino acids, and steroids through transzonal projections (TZPs) and gap junctions [9–11]. As the nucleolar configuration transitions from the non-surrounded (NSN) to the surrounded (SN), the oocyte undergoes transcriptional silencing and gains the competence to resume meiosis [6, 12]. The subsequent meiotic maturation relies on the translation of maternal RNAs stored in the oocyte cytoplasm [13, 14]. Therefore, spatial and temporal control of protein synthesis is crucial for oocyte growth and maturation.

Eukaryotic translation initiation factor 2B (eIF2B) complex comprises two copies of α, β, γ, δ, and ε subunits. Specifically, the enzyme’s catalytic center of eIF2B is located in the ε subunit, which closely associates with the γ subunit for cooperative function. The β and δ subunits form the structural core of the complex, bridged by the eIF2Bα subunit across the symmetry interface [15]. As a guanine nucleotide exchange factor (GEF) required for cytoplasmic protein synthesis, eIF2B activates its partner factor eIF2 to initiate translation [16]. In each translation cycle, eIF2 first assembles the ternary complex (TC) with GTP and Met-tRNAi [17]. Then, TC (eIF2·GTP·Met-tRNAi) binds to 40S ribosomal subunit, eIF1, eIF1A, eIF3 and eIF5 to form 43S preinitiation complex (PIC) [18]. The 43S-PIC is then recruited to the mRNA and scans the 5′ untranslated region (5′ UTR) to locate the initiation codon and initiate translation [19, 20]. After start codon recognition, the GTP bound to eIF2 is hydrolyzed by eIF5 to yield inactive eIF2·GDP, which is then released from the mRNA. A new cycle of mRNA translation begins as eIF2·GDP converts into eIF2·GTP, which binds Met-tRNAi to create a new TC [21, 22].

In humans, various translation initiation factor mutations are associated with the clinical onset of POI, such as *EIF2B* subunits, *EIF4ENIF1* (EIF4E nuclear import factor), and *EIF1AX* (EIF1AX-linked) [23–25]. However, the pathogenic causality and underlying mechanisms remain largely unknown. In mice, haploinsufficiency of *Eif4enif1* impairs mitochondrial dynamics by disrupting mitochondrial dynamics protein translation, resulting in female subfertility [26]. Deletion of *Eif4e1b* in oocytes leads to a 2-cell block and infertility by disrupting the translation of maternal mRNA [27]. In our previous research, the *Eif2s2* deletion in premeiotic germ cells leads to oocyte arrest at the early diplotene stage, and apoptosis via impairing homologous recombination and mitochondrial function, and *Eif5* depletion in oocytes causes apoptosis through reactive oxygen species (ROS) accumulation and the DNA damage pathway [28, 29]. Targeted mutation of *Eif2s3y* blocks spermatogonial differentiation and spermatogenesis, resulting in male infertility [30]. In addition, impaired function of several translation-related factors can also lead to female infertility. For example, DDB1 (DNA-binding protein 1) and CUL4 (cullin-ring finger ligase 4) associated factor 13 (DCAF13) participated in the 18S rRNA processing, which regulates translation initiation. Deletion of *Dcaf13* in oocytes stunts chromatin configuration transition by reducing global protein synthesis, leading to arrested oocyte development and female sterility [6]. Deletion of both zygote arrest-1 and −2 (*Zar1/2*) in germ cells impairs meiosis resumption and early embryonic development by hindering the translation of maternal mRNAs [31]. In short, this evidence suggests that deficiencies in different translational regulators can lead to various female fertility defects, including POI-like phenotype.

Mutations in any subunit of the *EIF2B* complex can lead to vanishing white matter disease (VWM), or termed childhood ataxia with central nervous system hypomyelination [32, 33]. Beyond this, the clinical manifestations of VWM also include growth retardation, renal hypoplasia, and ovarian dysfunction (primarily POI); this specific association has been termed ovarioleukodystrophy [32]. However, the role of eIF2B in maintaining ovarian function remains unclear. Here, conditional deletion of *Eif2b* subunits in primordial oocytes using *Gdf9*-Cre revealed the roles of eIF2B in oocyte growth and follicle development. The results showed that deleting *Eif2b2*, *Eif2b3*, *Eif2b4*, or *Eif2b5* in mouse oocytes caused oocyte apoptosis in early growing follicles, ultimately resulting in female infertility. Mechanistically, these phenotype defects were attributed to DNA damage and mitochondrial dysfunction in oocytes.

## Materials and methods

### Animals

The *Eif2b1*^flox/flox^, *Eif2b2*^flox/flox^, *Eif2b3*^flox/flox^, *Eif2b4*^flox/flox^, and *Eif2b5*^flox/flox^ mice were constructed by GemPharmatech Co., Ltd (Supplementary Fig. 1A), and the *Gdf9*-Cre mice on a C57BL/6J genetic background were obtained from The Jackson Laboratory. The *Eif2b1*, *Eif2b2*, *Eif2b3*, *Eif2b4*, and *Eif2b5* conditional knockout mice were generated by mating the *Eif2b1*^flox/flox^, *Eif2b2*^flox/flox^, *Eif2b3*^flox/flox^, *Eif2b4*^flox/flox^, and *Eif2b5*^flox/flox^ female mice with *Gdf9*-Cre (hereafter referred to as *Eif2b1-*, *Eif2b2-*, *Eif2b3-*, *Eif2b4-,* and *Eif2b5-*cKO) male mice. Genomic DNA from mice was used to identify the genotype by PCR, and primers for genotyping are shown in Table S1. All animal studies conducted were approved by the Institutional Animal Care and Use Committee of the South China University of Technology.

### Histomorphological examination and follicle counting

The ovaries collected from wild-type (WT), *Eif2b1-*, *Eif2b2-*, *Eif2b3-*, *Eif2b4-*, and *Eif2b5-*cKO mice at different ages were fixed in 4% paraformaldehyde (PFA, Solarbio, P1110) for 12 h. Subsequently, these ovaries were dehydrated, embedded, and sectioned. The 5-μm thick ovarian slices were further stained, and the number of primordial, primary, early secondary (2 layers of granulosa cells), late secondary (≥3 layers of granulosa cells), and antral follicles was counted following the method as previously described [27]. All follicle counts were performed by blinded observers.

### Oocyte collection and in vitro maturation

WT, *Eif2b1-*, *Eif2b2-*, *Eif2b3-*, *Eif2b4-*, and *Eif2b5-*cKO mice at postnatal day (PD) 21 received an injection of 5 IU equine chorionic gonadotropin (eCG). 48 h after PMSG injection, the germinal vesicle (GV) oocytes were separated and harvested from cumulus–oocyte complexes (COCs) in the ovaries. The GV oocytes were cultured in an M16 medium covered with mineral oil at 37 °C, and then the rates of germinal vesicle breakdown (GVBD) and first polar body (PB1) extrusion were calculated after culturing for 3 h and 14 h, respectively. For rescue experiments, GV oocytes from *Eif2b4-*cKO mice were divided into four groups: vehicle (0.1% DMSO, D2650, Sigma), AZD1390 (DNA damage signaling blockade, 3 nM, HY-109566, MCE), N-acetylcysteine (NAC, ROS scavenger, 2 mM, S0077, Beyotime), and Z-VAD-FMK (apoptosis inhibitor, 20 μM, HY-16658B, MCE). We examined the rates of oocytes with GVBD or PB1 extrusion at the end of 14 h culture. In some experiments, the female mice received an injection of 5 IU eCG, followed by 5 IU human chorionic gonadotropin (hCG) 48 h later. Finally, the metaphase II (MII) oocytes were harvested from COCs in the mouse oviducts 13 h post-hCG injection, and their quantity and quality were examined.

### Immunofluorescence staining

The GV oocytes collected from WT, *Eif2b1-*, *Eif2b2-*, *Eif2b3-*, *Eif2b4-*, and *Eif2b5-*cKO mice were successively placed in 4% PFA, 0.25% Triton X-100, and 3% BSA for fixation, permeabilization, and blocking. After these steps, the oocytes were incubated with the primary antibodies at 4 °C overnight, followed by incubation with fluorescent secondary antibodies at 37 °C for 1 h. Finally, the oocytes were counterstained with 4′, 6-diamidino-2-phenylindole (DAPI) and sealed in an anti-fluorescence quencher. The immunofluorescence staining of ovarian sections was performed following a procedure described previously [34]. Image acquisition of oocytes and ovarian sections was performed using an LSM 800 confocal microscope with the same parameters, and then the fluorescence intensity was measured by ZEN software (Carl Zeiss, Version 3.1). All positive signals were assessed by the blinded observers.

### Bromodeoxyuridine (BrdU) incorporation assay

The WT and *Eif2b4*-cKO mice received an injection of BrdU (100 mg/kg) 4 h before sacrifice. Subsequently, the ovaries were collected to prepare tissue sections, and the sections were subjected to immunofluorescence staining of BrdU. Finally, the percentage of granulosa cells with BrdU-positive signals in primary and secondary follicles of the largest five sections was calculated to assess granulosa cell proliferation.

### TUNEL assay

Apoptosis of ovarian cells was evaluated by Click-iT™ Plus TUNEL Assay (1982275, Thermo Fisher). In brief, the dewaxed and rehydrated ovarian sections were first fixed and permeabilized by 4% PFA and proteinase K, respectively, followed by incubation with a TUNEL reaction mixture. The sections were counterstained with DAPI after three washes with PBS, and the image acquisition was performed using an LSM 800 confocal microscope. The TUNEL-positive signals in each section were counted.

### Puromycin incorporation assay

The puromycin incorporation was performed to assess the translation efficiency. Briefly, the WT, *Eif2b1*-, and *Eif2b4*-cKO mice were injected with puromycin (65 mg/kg) 1.5 h before sacrifice. Subsequently, the GV oocytes were used to extract the total protein for Western blot analysis using anti-puromycin primary antibody. The relative gray value of the anti-puromycin signal (normalized to β-actin) was calculated from the total protein lanes using ImageJ software.

### RNA isolation and analysis

Total RNA of WT and *Eif2b4*-cKO GV oocytes was extracted using a commercial kit (74004, Qiagen), and then the RNA was used to create cDNA using a reverse transcription kit (205311, Qiagen). qRT-PCR was performed to quantify the mRNA levels using a LightCycler 96 system (Roche). The relative mRNA level was calculated based on the 2^−ΔΔCt^ method, with ribosomal protein L19 (*Rpl19*) serving as an endogenous control. To perform RNA sequencing (RNA-seq) analysis, the GV oocytes were collected from WT and *Eif2b4*-cKO mice at PD21. All samples were analyzed by NeoRibo Biotechnology Co., Ltd (Hangzhou, China). All primers for qRT-PCR are shown in Table S2.

### Western blot

In all, 100 GV oocytes collected from each genotype were lysed in a RIPA buffer to extract total protein. Subsequently, the protein samples underwent separation with an SDS-PAGE gel and were transferred to a PVDF membrane. After blocking with 5% skim milk, the membranes were incubated with primary antibodies at 4 °C overnight, followed by incubation with appropriate secondary antibodies (1:5000, ZSGB-BIO). Finally, all membranes were exposed to Immobilon Western Chemiluminescent HRP substrate (Millipore), and bands were detected using a Tanon 5200 imaging system. Relative gray values of protein bands were calculated using ImageJ software, with β-actin serving as the internal control. All primary antibodies are detailed in Table S3.

### Ribosome profiling sequencing (Ribo-seq) analysis

For Ribo-seq analysis, ribosomes were extracted from 2000 GV oocytes of each genotype and then analyzed by NeoRibo Biotechnology Co., Ltd (Hangzhou, China). In brief, the oocytes were lysed under conditions that preserved polysomes, and ribosome–mRNA complexes were digested with RNase I to generate ribosome-protected fragments (RPFs). The fragments were then purified, dephosphorylated, ligated to adapters, and converted into cDNA libraries. The resulting libraries were quantified, pooled, and subjected to high-throughput sequencing on the Illumina NovaSeq X Plus platform using a paired-end 150 bp (PE150) strategy. Raw sequencing reads were subjected to quality trimming and ribosomal RNA (rRNA) depletion, and were aligned to the transcriptome using Cutadapt, Bowtie, and STAR, respectively. Downstream ribosome footprint analyses (read length distribution, P-site offset, 3-nt periodicity, metagene occupancy, and cross-condition comparisons) were then performed with the RiboWaltz package in R following its default workflow.

### Active mitochondrial staining

GV oocytes from WT and *Eif2b4*-cKO mice were incubated with 100 nM MitoTracker Deep Red FM for 30 min at 37 °C, followed by washing with preheated M2 medium. Subsequently, these oocytes were counterstained with Hoechst 33342 and submitted to a confocal microscope for image acquisition.

### Measurement of mitochondrial membrane potential

GV oocytes from WT and *Eif2b4*-cKO mice were incubated with JC1 for 30 min at 37 °C (C2003S, Beyotime), followed by washing with preheated M2 medium. Finally, the image acquisition of living oocytes was performed using an LSM 800 confocal microscope, and mitochondrial membrane potential (MMP) is indicated by a ratio of red fluorescence intensity (J-aggregates) to green fluorescence intensity (J-monomers).

### Detection of reactive oxygen species (ROS)

The total ROS levels of GV oocytes from WT and *Eif2b4*-cKO mice were detected by commercial kits (S0033S, Beyotime). Briefly, the oocytes were incubated in a serum-free medium with 10 μM DCFH-DA for 30 min at 37 °C for ROS detection. Finally, all oocytes were submitted to a confocal microscope for image acquisition and analysis.

### Detection of ATP content

The ATP content of oocytes from WT and *Eif2b4*-cKO mice was detected with a commercial kit (C2003S, Beyotime). Specifically, 30 GV oocytes of each genotype were transferred into a lysis buffer and incubated for 10 min on ice. After centrifugation, 10 μL of supernatant and gradient concentration of the standards were submitted to a microplate reader to measure the optical density value. Finally, the ATP content was calculated based on the standard curve generated with the standards.

### Mitochondrial DNA (mtDNA) copy number measurement

The measurement of mtDNA copy number was conducted as previously described [35]. Total genomic DNA from WT and *Eif2b4*-cKO GV oocytes was extracted using the TIANamp Genomic DNA kit (TIANGEN, Beijing, China). qRT-PCR using SYBR green with primers specific (Table S4) to the mitochondrial and nuclear genomes to calculate mitochondrial-to-nuclear DNA (mtDNA/nDNA) ratio, thereby quantifying the mtDNA copy number.

### Co-culture of granulosa cells and oocytes

Granulosa cells (GCs) were isolated from PD21 mice and cultured in the 35 mm glass-bottom dishes. After overnight attachment in 1 mL DMEM/F12 medium with 10% fetal bovine serum, the culture medium was removed. Then, 20 denuded GV oocytes from WT or *Eif2b4*-cKO mice were added into each dish, and co-cultured in serum-free DMEM/F12 supplemented with 0.3% BSA and 2 μM milrinone (for GV arrest). After 48 h of co-culture, granulosa cell proliferation was evaluated by PCNA immunofluorescence, and the percentage of PCNA-positive cells was quantified.

### Statistical analysis

The experimental data are presented as the mean ± standard deviation (SD), with a minimum of three biological replicates performed for each experiment. To assess significance and visualize the data, this study employed the Student’s *t* test along with GraphPad Prism version 8.3.

## Results

### Oocyte-expressed eIF2B subunits are indispensable for follicle development and female fertility

**Fig. 1:**
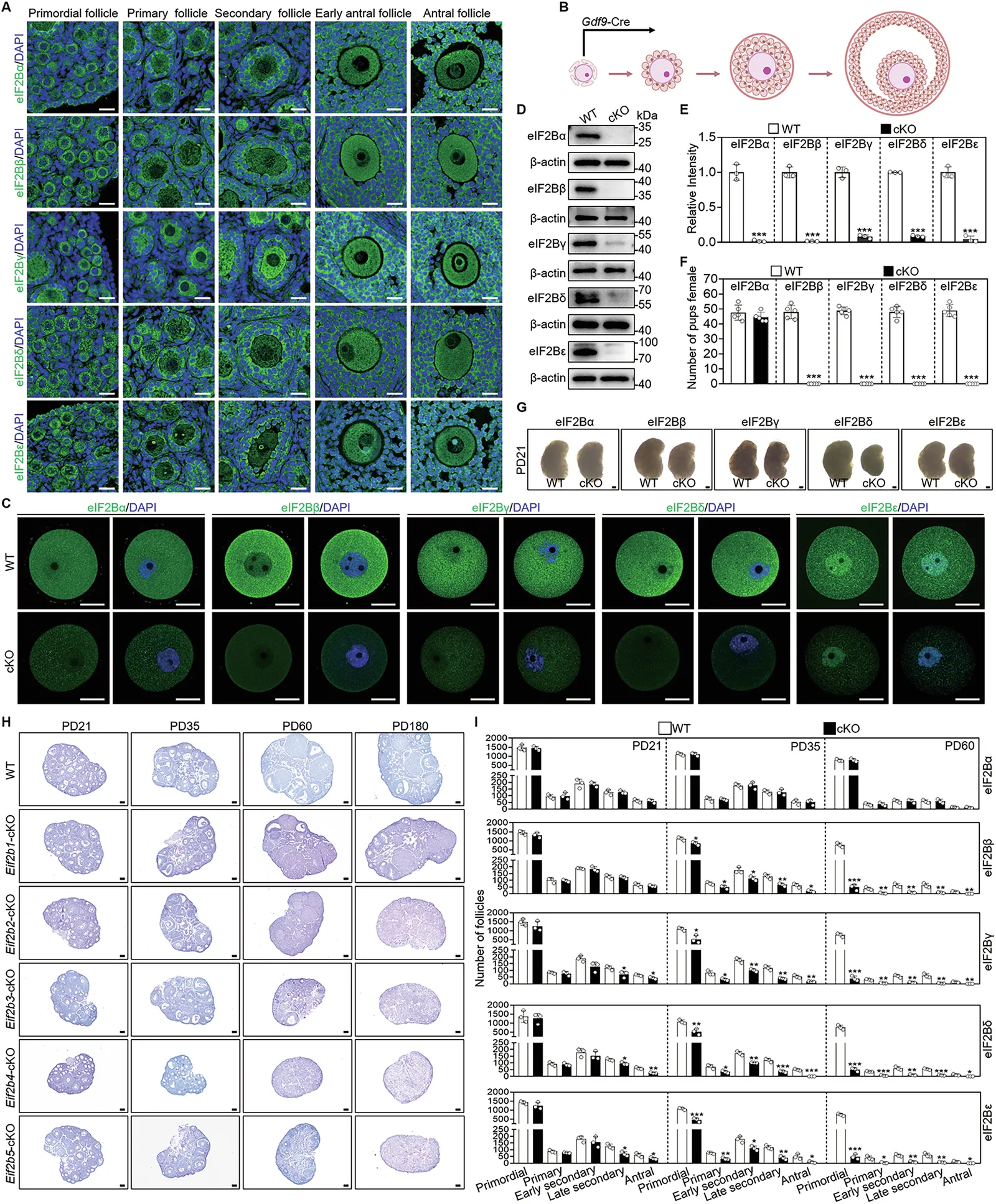
Deletion of *Eif2b* subunits in oocytes impairs follicle development and female fertility. **A** Representative image of eIF2Bα, eIF2Bβ, eIF2Bγ, eIF2Bδ, and eIF2Bε staining for follicles at different stages. Cell nuclei were visualized using DAPI. **B** Schematic diagram showing oocyte-specific deletio*n* of *Eif2b* subunits in primordial follicle by *Gdf9*-Cre (cKO). **C** Immunofluorescence staining of eIF2Bα, eIF2Bβ, eIF2Bγ, eIF2Bδ, and eIF2Bε in WT and cKO oocytes at PD21. **D**, **E** Western blot analysis of eIF2Bα, eIF2Bβ, eIF2Bγ, eIF2Bδ, and eIF2Bε protein levels in WT and cKO. **F** Cumulative numbers of pups per WT and cKO (*n* = 5 females for each genotype) mice during the 40 weeks of a fertility test. **G** Representative images of ovaries isolated from WT and cKO female mice at postnatal day 21 (PD21). **H** PAS staining showing ovarian histology of WT and cKO female mice. **I** Counting of follicle number at different age stages, with *n* = 3 females each genotype. In each experiment, *n* ≥ 3 biological replicates. Bars indicate the mean ± SD. A two-sided Student’s *t* test was used to determine *P* values. (*P < 0.05, **P < 0.01 and ***P < 0.001). Scale bar: 25 μm (**A**, **C**), and 100 μm (**H**).

Immunofluorescence staining showed that eIF2Bα, eIF2Bβ, eIF2Bγ, eIF2Bδ, and eIF2Bε were expressed in granulosa cells and oocytes all follicle stages (Fig. 1A). Therefore, we generated *Eif2b1-*, *Eif2b2-*, *Eif2b3-*, *Eif2b4-*, and *Eif2b5-*cKO mice to investigate the role of eIF2Bα, eIF2Bβ, eIF2Bγ, eIF2Bδ, and eIF2Bε in follicle development (Fig. 1B and Supplementary Fig. 1A). The knockout efficiency was validated by immunofluorescence and western blot (Fig. 1C–E). Fertility testing showed that the depletion of *Eif2b2, Eif2b3, Eif2b4* and *Eif2b5* resulted in complete female sterility, whereas the depletion of *Eif2b1* did not affect female fertility (Fig. 1F). Morphological observation showed that the ovarian size was significantly smaller in *Eif2b4-*cKO mice than in WT mice at PD21 (Fig. 1G and Supplementary Fig. 1B). PAS staining and follicle counting showed that *Eif2b1* depletion did not affect follicle development, whereas the depletion of *Eif2b2*, *Eif2b3*, *Eif2b4* and *Eif2b5* resulted in follicle development defects of varying severity, with that of *Eif2b4* causing the most profound impairment (Fig. 1H, I). The number of late secondary and antral follicles, but not the number of primordial, primary, and early secondary follicles, in *Eif2b4*-cKO mice ovaries was significantly decreased at PD21 compared with the corresponding WT mice ovaries. At PD35, the number of all stage follicles was significantly decreased in *Eif2b4*-cKO mice ovaries. Compared with the corresponding WT mice, the mature follicles were not observed in *Eif2b4*-cKO ovaries at PD35, suggesting that the *Eif2b4*-cKO mice undergo premature ovarian failure as early as PD35. Furthermore, all follicles were exhausted in *Eif2b4*-cKO mice at PD180 (Fig. 1H and Supplementary Fig. 1C). These findings suggest that the depletion of *Eif2b2*, *Eif2b3*, *Eif2b4*, or *Eif2b5* in oocytes results in follicle development defects, ultimately leading to female infertility.

### Depletion of *Eif2b4* in oocytes causes defects in oocyte meiotic maturation

**Fig. 2:**
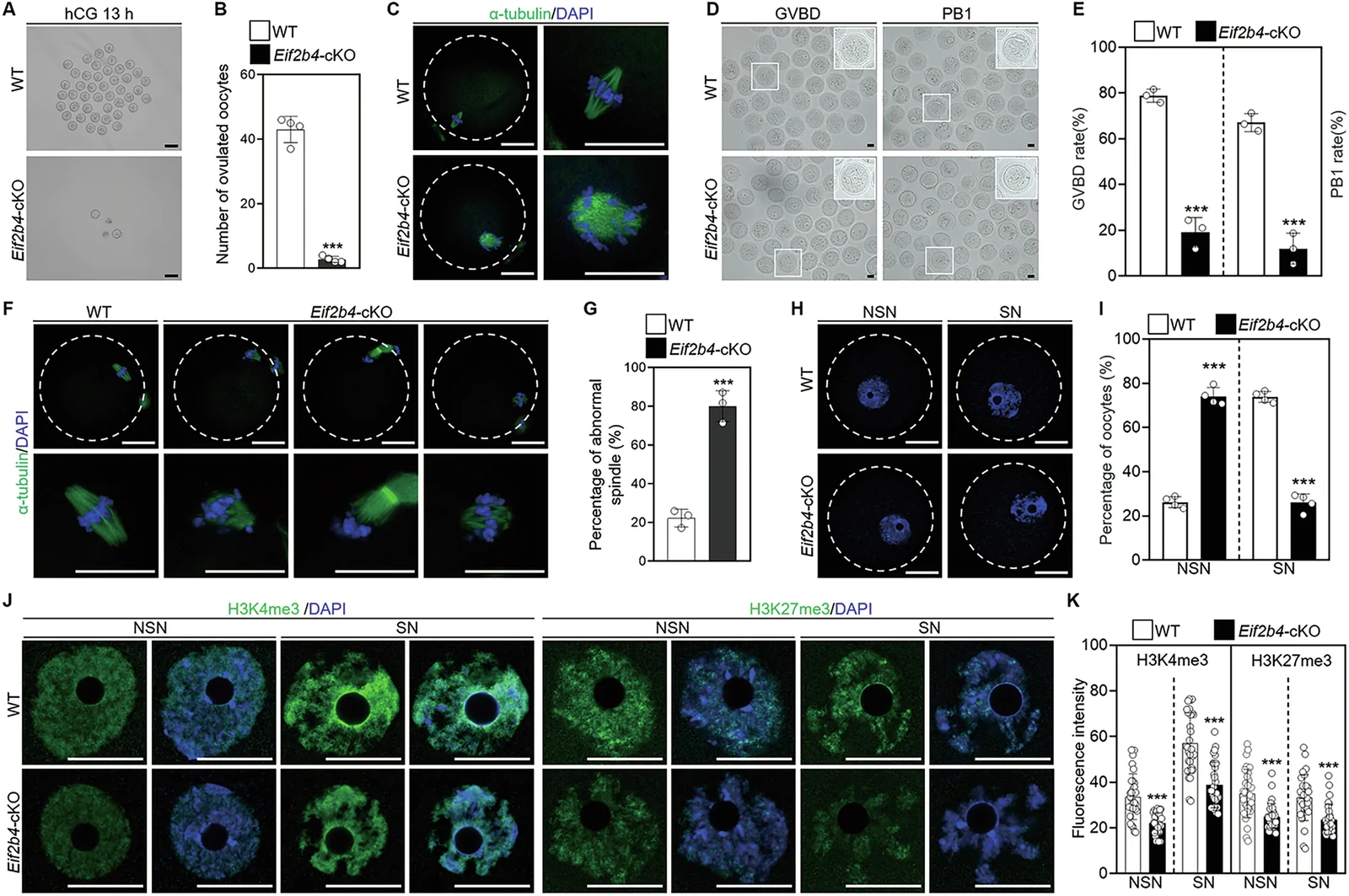
*Eif2b4* deletion in oocytes impairs oocyte maturation. **A** Representative image of ovulated oocytes 13 h post-human chorionic gonadotropin (hCG) treatment. **B** Number of ovulated oocytes per female mouse, *n* = 4 females each genotype. **C** α-tubulin staining showing spindle assembly in superovulated oocytes from WT and cKO mice. **D**, **E** Representative images and the percentage of germinal vesicle breakdown (GVBD) and first polar body (PB1) extrusion in WT and cKO oocytes cultured in vitro. *n* = 3, and 67–102 oocytes were analyzed in each repetition. **F** α-tubulin staining showing spindle assembly in oocytes cultured in vitro from WT and cKO mice. **G** The percentage of abnormal spindle assembly in oocytes cultured in vitro from WT and cKO mice. **H** DAPI staining of the oocytes with non-surrounded nucleolus (NSN) and surrounded nucleolus (SN) chromatin configurations in WT and cKO mice. **I** The percentage of NSN and SN type oocytes in WT (*n* = 203 oocytes) and cKO (*n* = 160 oocytes) mice. **J** Immunofluorescence staining of H3K4me3 and H3K27me3 in WT and cKO oocytes. **K** Quantification of H3K4me3 (*n* = 30 oocytes) and H3K27me3 (*n* = 30 oocytes) fluorescence intensity. In each experiment, *n* ≥ 3 biological replicates. Bars indicate the mean ± SD. A two-sided Student’s *t* test was used to determine *P* values. (***P < 0.001). Scale bar: 100 μm (**A**) and 25 μm (**C**, **D**, **F**, **H**, **J**).

The *Eif2b4*-cKO mice were selected to assess the effects on oocyte developmental competence, as mice lacking other eIF2B subunits exhibited similar reproductive phenotypes. The results of superovulation showed that oocyte number was significantly decreased in *Eif2b4*-cKO mice compared with that in WT mice (3 vs 43; Fig. 2A–C). In vitro maturation of fully grown oocytes (FGOs) from *Eif2b4*-cKO mice showed that both the GVBD and PB1 extrusion rates were significantly decreased (19.10% vs 78.77% and 11.97% vs 67.02%, respectively; Fig. 2D, E). Further analysis revealed that these oocytes with PB1 exhibited a significantly increased percentage of abnormal spindles compared with those of WT oocytes (80.09% vs 22.26%; Fig. 2F, G). On the other hand, the *Eif2b4* deletion oocyte defects observed in vitro maturation may be partially attributable to the impaired follicle development. Furthermore, the FGOs collected from *Eif2b4*-cKO and WT mice were also assessed. Analysis of chromatin configuration showed that 73.81% of the oocytes were SN in WT mice, while only 25.94% of the oocytes were SN in *Eif2b4*-cKO mice (Fig. 2H, I). Consistent with this, the accumulation of H3K4 trimethylation (H3K4me3), which is indispensable for chromatin configuration transition from NSN to SN [36], was significantly decreased in *Eif2b4*-cKO oocytes compared with that in WT oocytes (Fig. 2J, K and Supplementary Fig. 2A). The accumulation of H3K27 trimethylation (H3K27me3) was also decreased in *Eif2b4*-cKO oocytes (Fig. 2J, K and Supplementary Fig. 2B), suggesting that nuclear development defects are present in these oocytes. These findings suggest that *Eif2b4* deletion in oocytes causes defects in nuclear maturation.

### Depletion of *Eif2b4* in oocytes causes defects in oocyte-granulosa cell communication

**Fig. 3:**
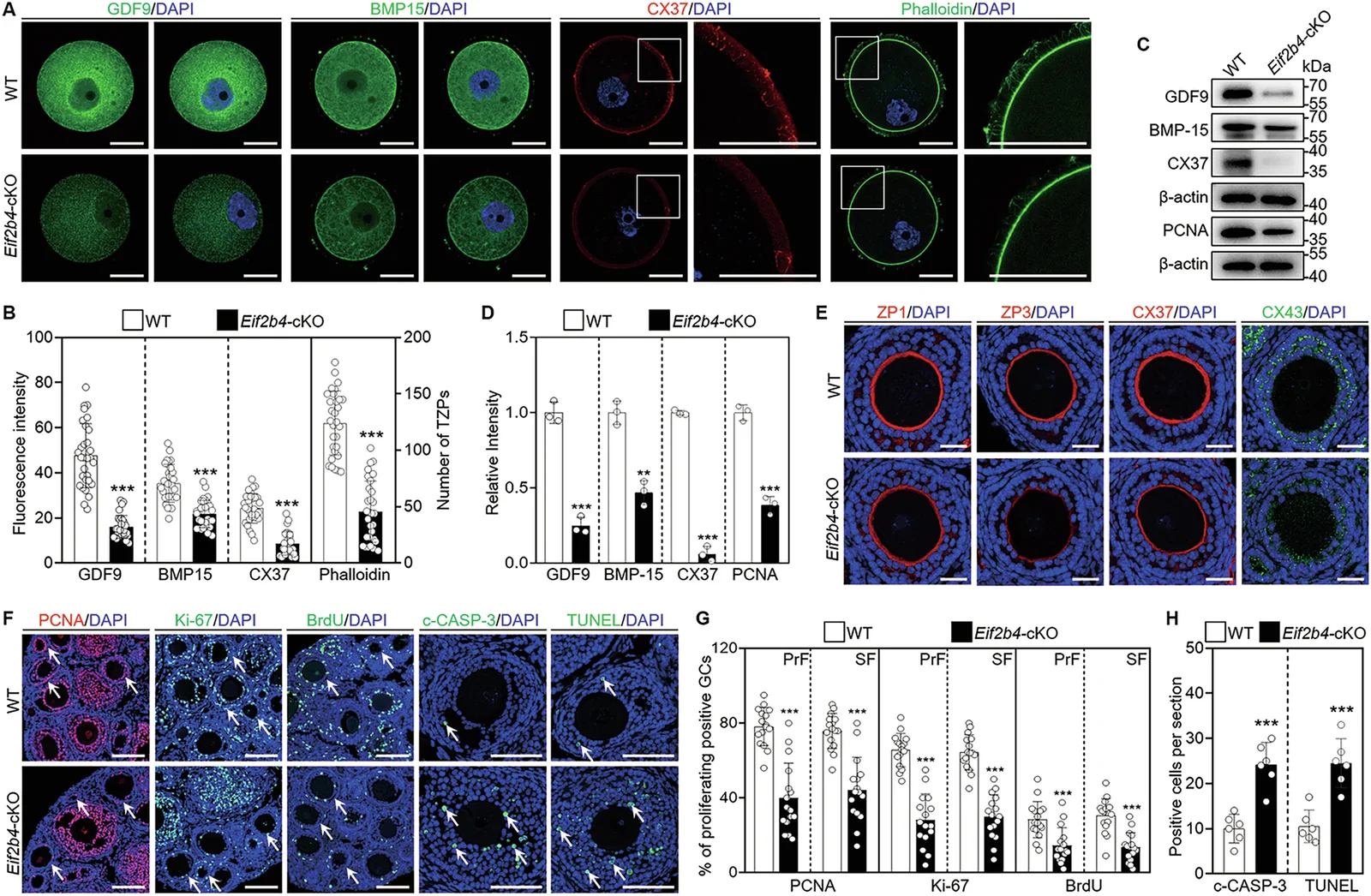
*Eif2b4* deletion in oocytes impairs granulosa cell proliferation and induces apoptosis. **A** Immunofluorescence staining of GDF9, BMP15, CX37, and Phalloidin in WT and cKO oocytes. **B** Quantification of fluorescence intensity and transzonal projections (TZPs) number in WT and cKO oocytes (*n* = 30). **C**, **D** Western blot analysis of GDF9, BMP15, and CX37 levels in WT and cKO oocytes, and PCNA levels in WT and cKO granulosa cells. **E** Immunofluorescence staining of ZP1, ZP3, CX37, and CX43 in WT and cKO ovaries. **F** Immunofluorescence staining of PCNA, Ki-67, BrdU, c-CASP-3 (cleaved Caspase-3), and TUNEL in WT and cKO ovaries at PD21. **G** The percentage of PCNA-, Ki-67- and BrdU-positive granulosa cells in primary follicles (PrF) and secondary follicles (SF). **H** Quantification of c-CASP-3- and TUNEL-positive granulosa cells in WT and cKO ovaries at PD21. In each experiment, *n* ≥ 3 biological replicates. Bars indicate the mean ± SD. A two-sided Student’s *t* test was used to determine *P* values. (**P < 0.01 and ***P < 0.001). Scale bar: 25 μm (**A**, **E**) and 100 μm (**F**).

Follicle development defect is closely associated with impaired bidirectional oocyte-granulosa cell communication [37]. Immunofluorescence staining and Western blot analysis showed that the protein levels of GDF9, BMP15, and connexin 37 (CX37) in *Eif2b4*-cKO oocytes were significantly decreased compared with those of WT oocytes (Fig. 3A–D). Moreover, the phalloidin staining showed a disorganized distribution and a decreased number of F-actin bundles in the zona pellucida (ZP) area of *Eif2b4*-cKO oocytes compared with WT oocytes (Fig. 3A, B). Immunofluorescence staining of ZP1, ZP3, CX37, and CX43 also revealed thinner ZPs in *Eif2b4*-cKO oocytes (Fig. 3E and Supplementary Fig. 3A, B).

To further reveal the causes of follicle development defects, Western blot analysis, immunofluorescence staining, and TUNEL were performed to assess the proliferation and apoptosis of granulosa cells. Experimental results showed a significant decrease in the percentage of granulosa cells positive for PCNA, Ki-67, or BrdU in the primary and secondary follicles (Fig. 3F, G and Supplementary Fig. 3B, C), alongside a significant increase in the number of granulosa cells positive for TUNEL or cleaved caspase-3 (c-CASP-3) in the secondary follicles of *Eif2b4*-cKO mice compared with those of WT mice (Fig. 3F, H and Supplementary Fig. 3C). Consistently, the protein levels of PCNA were significantly decreased in the granulosa cells of *Eif2b4*-cKO mice (Fig. 3C, D). Co-culture experiments were performed using WT granulosa cells with WT or *Eif2b4*-cKO oocytes. The results showed that *Eif2b4*-cKO oocyte co-culture resulted in the decreased the percentage of PCNA-positive cells in WT granulosa cells compared with that in WT oocyte co-culture (Supplementary Fig. 3D, E). In short, the depletion of *Eif2b4* in oocytes leads to secondary granulosa cell proliferation defects and apoptosis, potentially due to impaired bidirectional communication.

### Depletion of *Eif2b4* in oocytes causes transcription and translation dysregulation

**Fig. 4:**
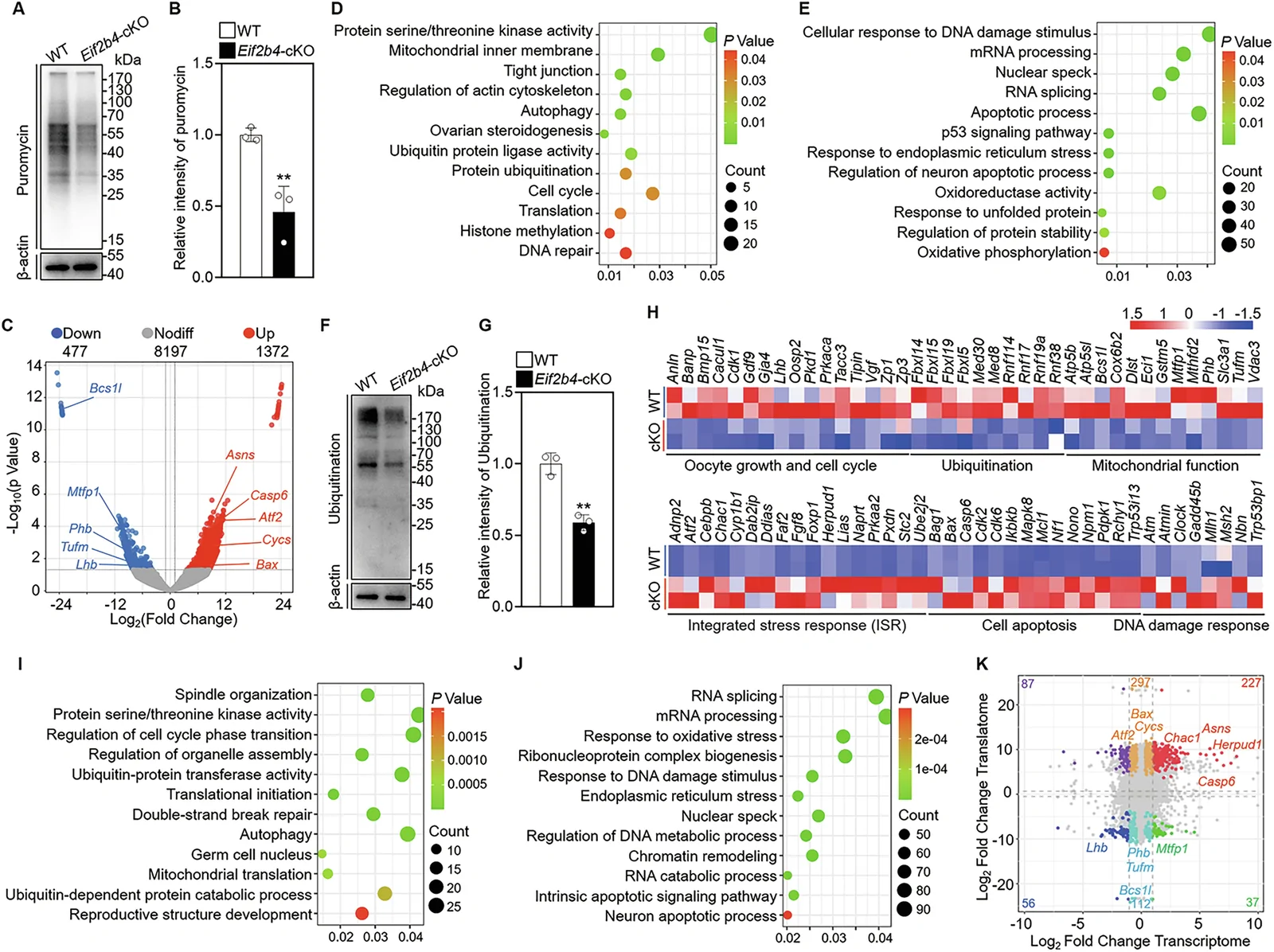
*Eif2b4* deletion in oocytes inhibits global protein translation. **A**, **B** Western blot analysis of puromycin incorporation in WT and cKO oocytes. **C** Volcano plot of differentially translated genes (DTGs) between WT and cKO oocytes. **D**, **E** Bubble chart illustrating the GO terms enriched by downregulated and upregulated DTGs in cKO oocytes. **F**, **G** Western blot analysis of the total ubiquitination levels in WT and cKO oocytes. **H** Heatmaps illustrating a group of downregulated and upregulated DTGs involved in the indicated biological processes in WT and cKO oocytes. **I**, **J** Bubble chart illustrating the GO terms enriched by downregulated and upregulated differentially translated efficiency genes (DTEGs) in cKO oocytes. **K** Nine-quadrant analysis of transcriptome and translatome in WT and cKO oocytes. Dashed lines indicate thresholds for transcriptome (FC ≥ 2.0) and translatome (FC ≥ 1.5). Bars indicate the mean ± SD. A two-sided Student’s *t* test was used to determine *P* values. (**P < 0.01).

eIF2B plays a crucial role in translation initiation by mediating the guanine nucleotide exchange on eIF2. Therefore, the translational efficiency was first detected using the puromycin incorporation method. Western blot analysis showed that the anti-puromycin intensity was significantly decreased in *Eif2b4*-cKO oocytes compared with WT oocytes (Fig. 4A, B), suggesting a decrease in the synthesis of new proteins. However, the anti-puromycin intensity was not changed in *Eif2b1*-cKO oocytes compared with that in WT oocytes (Supplementary Fig. 4A, B). Furthermore, we performed Ribo-seq and RNA-seq using oocytes from *Eif2b4*-cKO and WT mice. Ribo-seq analysis revealed the identification of a total of 1849 differentially translated genes (DTGs) (Fig. 4C and Supplementary Fig. 4C, D). Compared with those of WT oocytes, 477 DTGs were decreased, and 1372 DTGs were increased by >1.5-fold change in *Eif2b4*-cKO oocytes (Fig. 4C). Gene Ontology (GO) analysis of the downregulated DTGs revealed processes such as ovarian steroidogenesis, cell cycle, mitochondrial function, tight junction, ubiquitination, and autophagy (Fig. 4D). Consistently, Western blot analysis revealed that the total ubiquitination level was significantly decreased in the *Eif2b4*-cKO oocytes compared with that in WT oocytes (Fig. 4F, G). The significant downregulation of DTGs such as *Bcs1l* (for mitochondrial respiratory chain complex assembly), *Tufm* (for mitochondrial protein translation), and *Mtfp1* (regulation of mitochondrial dynamics (Fig. 4C, H) could lead to mitochondrial dysfunction in *Eif2b4*-cKO oocytes. The upregulated DTGs were enriched in processes such as mRNA processing, DNA damage, oxidative stress, and cell apoptosis (Fig. 4E). In particular, the significant upregulation of DTGs, such as *Atm*, *Nbn*, and *Trp53bp1*, may trigger the DNA damage response (DDR) in *Eif2b4*-cKO oocytes. Furthermore, the upregulation of proapoptotic genes, such as *Bax*, *Casp6*, and *Cycs*, could directly induce apoptosis (Fig. 4C, H). Conducting matched Ribo-seq and RNA-seq analysis revealed 3249 differentially translated efficiency genes (DTEGs) (696 downregulation and 2553 upregulation) in the oocytes of *Eif2b4*-cKO mice (Supplementary Fig. 4E). Consistent with GO enrichment analysis of DTGs, the downregulated DTEGs were also related to cell cycle, autophagy, protein ubiquitination and translation initiation, while the upregulated DTEGs were related to oxidative stress, endoplasmic reticulum stress, DNA damage and apoptosis process (Fig. 4I, J). These results indicate that the overall changes in translation levels are partially attributed to the changes in translation efficiency, and these impaired functions may result in developmental defects in *Eif2b4*-cKO oocytes.

Subsequently, integrated analysis of transcriptome and translatome was performed using a nine-quadrant diagram (Fig. 4K and Supplementary Fig. 4F). The results showed that 409 genes exhibited differential changes at the translational level only. These included genes involved in mitochondrial function (such as *Phb*, *Tufm*, and *Bcs1l*), DDR (such as *Atm*, *Gadd45b*, and *Trp53bp1*), and apoptosis (such as *Bax*, *Cycs*, and *Atf2*). Some integrated stress response (ISR)-related genes, such as *Asns*, *Chac1*, and *Herpud1*, are upregulated at both transcriptional and translational levels in *Eif2b4*-cKO oocytes (Fig. 4K). Notably, the translation efficiency of several follicle development-related genes (such as *Gdf9*, *Bmp15*, *Oosp2*, *Hmgb2*, and *Apc*) was significantly downregulated, while that of DNA damage-related genes (such as *Atm*, *Nbn*, and *Trp53bp1*) was significantly upregulated. In addition, integrated analysis also showed that ISR-related factors (such as *Asns*, *Chac1*, and *Cebpb*) in *Eif2b4*-cKO oocytes are significantly increased in translation efficiency and transcription level (Supplementary Fig. 4F). These findings further indicate that depletion of *Eif2b4* in oocytes induces ISR activation and DDR.

**Fig. 5:**
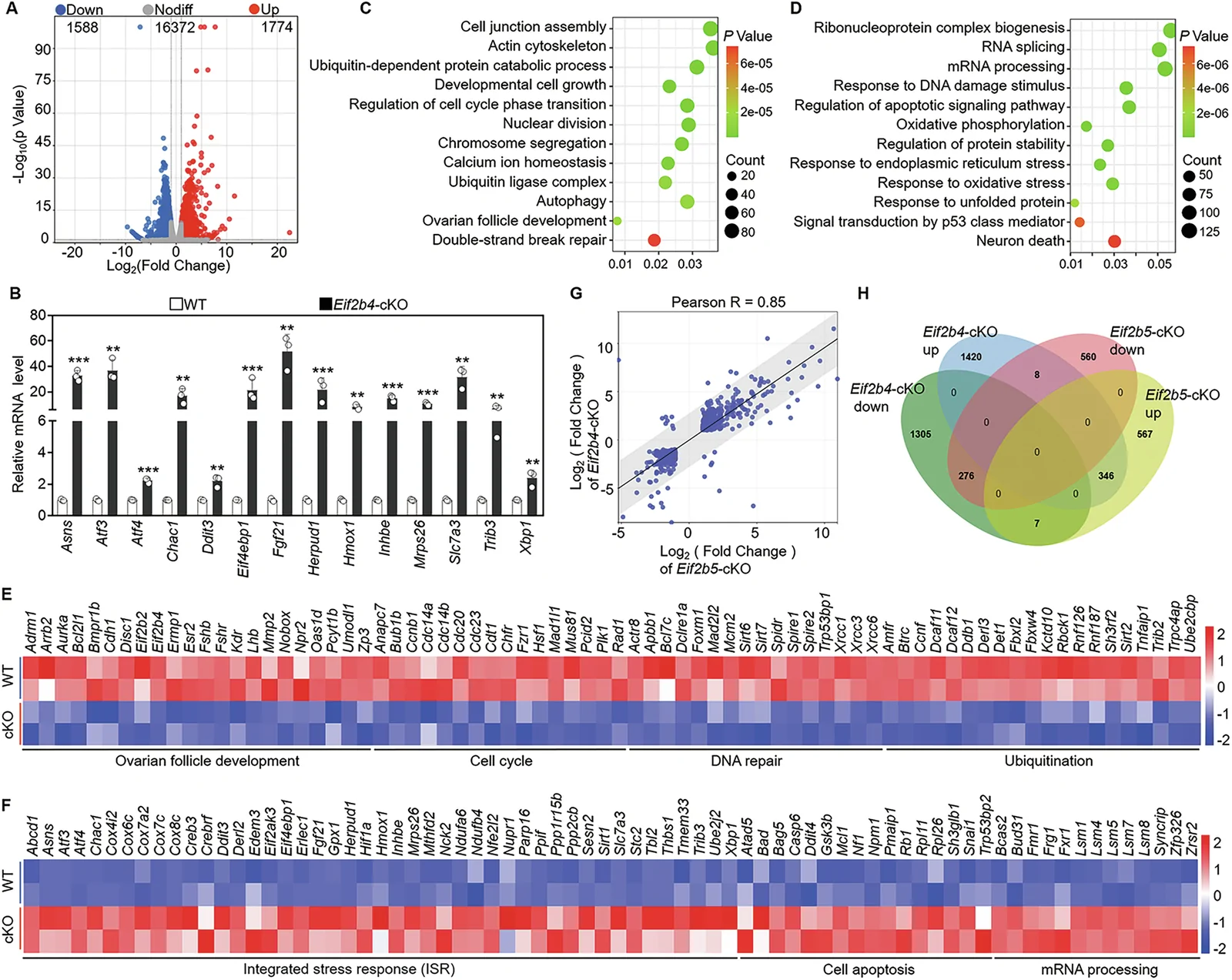
*Eif2b4* deletion in oocytes impairs the integrity of the transcriptome. **A** Scatter plot showing the transcripts with differential expression in WT and cKO oocytes. **B** qRT-PCR validating the differential transcripts identified by RNA-seq. **C**, **D** Bubble chart illustrating the GO terms enriched by downregulated and upregulated transcripts in cKO oocytes. **E**, **F** Heatmaps illustrating a group of downregulated and upregulated transcripts involved in the indicated biological processes in WT and cKO oocytes. **G** Scatter plot showing the correlation of differential transcripts between *Eif2b4-*cKO and *Eif2b5-*cKO oocytes. **H** Venn diagram showing the relationship of differential transcripts in *Eif2b4-*cKO and *Eif2b5-*cKO oocytes. Bars indicate the mean ± SD. A two-sided Student’s *t* test was used to determine *P* values. (**P < 0.01 and ***P < 0.001).

Compared with those of WT oocytes, 1588 transcripts were decreased, and 1774 transcripts were increased by >two fold change in *Eif2b4*-cKO oocytes (Fig. 5A). The qRT-PCR verification was performed on the candidate transcripts (Fig. 5B). Like transcriptomic results, GO analysis of downregulated transcripts revealed processes such as follicle development, ubiquitination, cell cycle phase transition and calcium ion homeostasis (Fig. 5C, E). The upregulated transcripts revealed processes such as ISR, DNA damage, cell apoptosis, and response to unfolded protein (Fig. 5D, F). Especially, the ISR-related genes, such as *Atf4*, *Asns*, *Chac1*, *Ddit3*, *Fgf21*, *Trib3*, and *Xpb1*, were significantly upregulated in *Eif2b4*-cKO oocytes compared with those in WT oocytes. Furthermore, the differentially expressed transcripts in *Eif2b4*-cKO oocytes showed a significant positive correlation (*R* = 0.85) with those in *Eif2b5*-cKO oocytes (Fig. 5G, H and Supplementary Fig. 5A–C). These results indicate that the translation inhibition in oocytes caused by the depletion of *Eif2b4* also results in transcription dysregulation.

### Mitochondrial dysfunction in the oocytes of *Eif2b4*-depletion mice

**Fig. 6:**
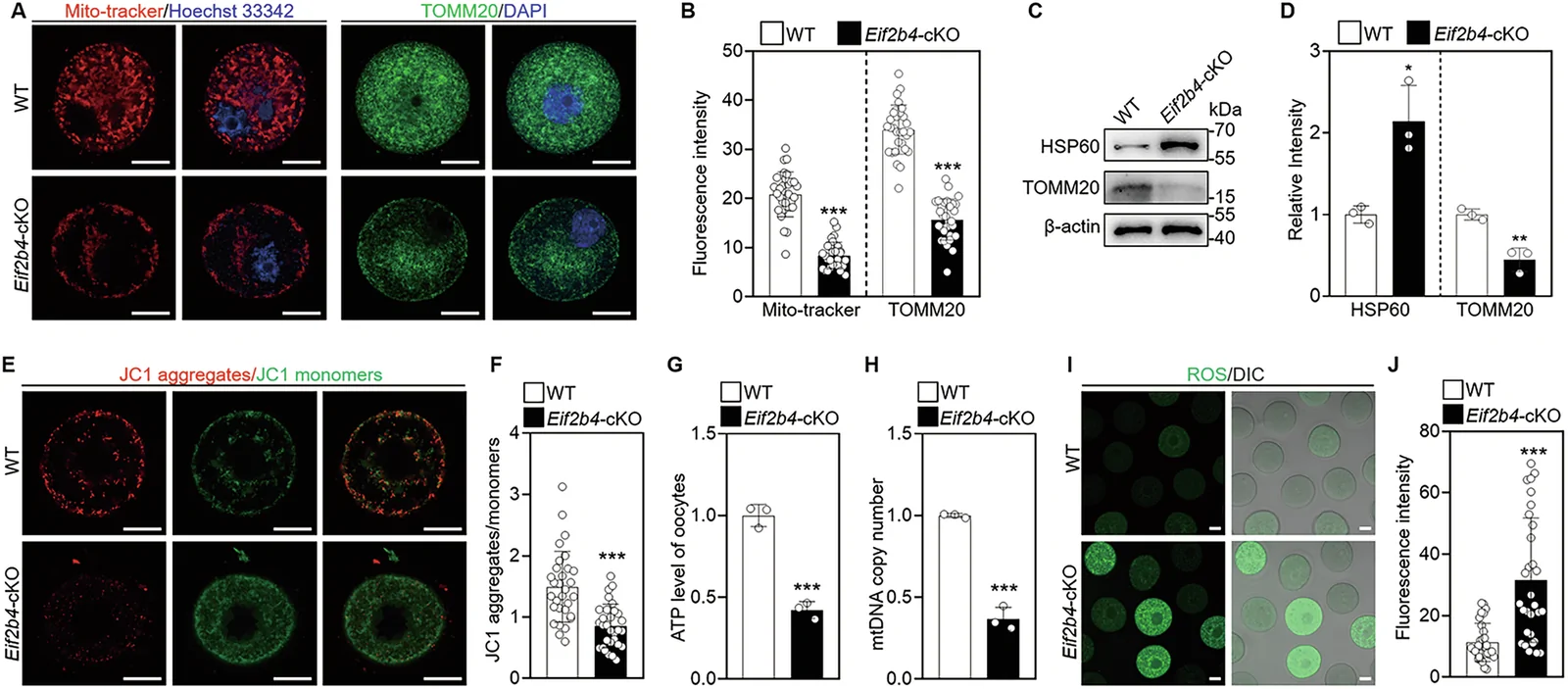
*Eif2b4* deletion in oocytes impairs mitochondrial function. **A** Representative image of MitoTracker and TOMM20 staining in WT and cKO oocytes. **B** Quantification of fluorescence intensity of MitoTracker and TOMM20 in WT and cKO oocytes (*n* = 30). **C**, **D** Western blot analysis of HSP60 and TOMM20 levels in WT and cKO oocytes. **E** Representative images of JC-1 staining. **F** Bar chart illustrating the red/green fluorescence ratio of JC-1 in WT and cKO oocytes (*n* = 30). **G** Quantitative analysis of the relative ATP levels in WT and cKO oocytes, *n* = 45 oocytes in each group. **H** Measurement of the mitochondrial DNA (mtDNA) copy number in WT and cKO oocytes, *n* = 100 oocytes in each group. **I** Representative images of reactive oxygen species (ROS) detected by DCFH staining in WT and cKO oocytes. **J** Quantification of ROS fluorescence intensity in WT and cKO oocytes (*n* = 30). In each experiment, *n* ≥ 3 biological replicates. Bars indicate the mean ± SD. A two-sided Student’s *t* test was used to determine *P* values. (*P < 0.05, **P < 0.01 and ***P < 0.001). Scale bar: 25 μm.

RNA-seq and Ribo-seq data demonstrate ISR activation in *Eif2b4*-cKO oocytes. Consequently, we examined the effect of *Eif2b4*-cKO on oocyte mitochondrial function. Mitochondrial staining showed that the mitochondria aggregated beneath the cell membrane, and MitoTracker fluorescence intensity was significantly decreased in *Eif2b4*-cKO mice compared with that of WT mice (Fig. 6A, B). Furthermore, compared with WT oocytes, the fluorescence intensity and protein levels of TOMM20 (a marker for the mitochondrial outer membrane) were significantly decreased, and the protein levels of heat shock protein 60 (HSP60, a molecular stress protein predominantly localized to the mitochondrion) were significantly increased in *Eif2b4*-cKO oocytes (Fig. 6A–D). Mitochondrial function analysis also showed that the mitochondrial membrane potential (JC1 aggregates/JC1 monomers), ATP levels, and mtDNA copy numbers were significantly decreased (Fig. 6E–H), while the ROS levels were significantly accumulated in *Eif2b4*-cKO oocytes compared with those in WT oocytes (Fig. 6I, J). These results indicate that the depletion of *Eif2b4* in oocytes results in mitochondrial dysfunction.

### Depletion of *Eif2b4* in oocytes triggers DNA damage and oocyte apoptosis

**Fig. 7:**
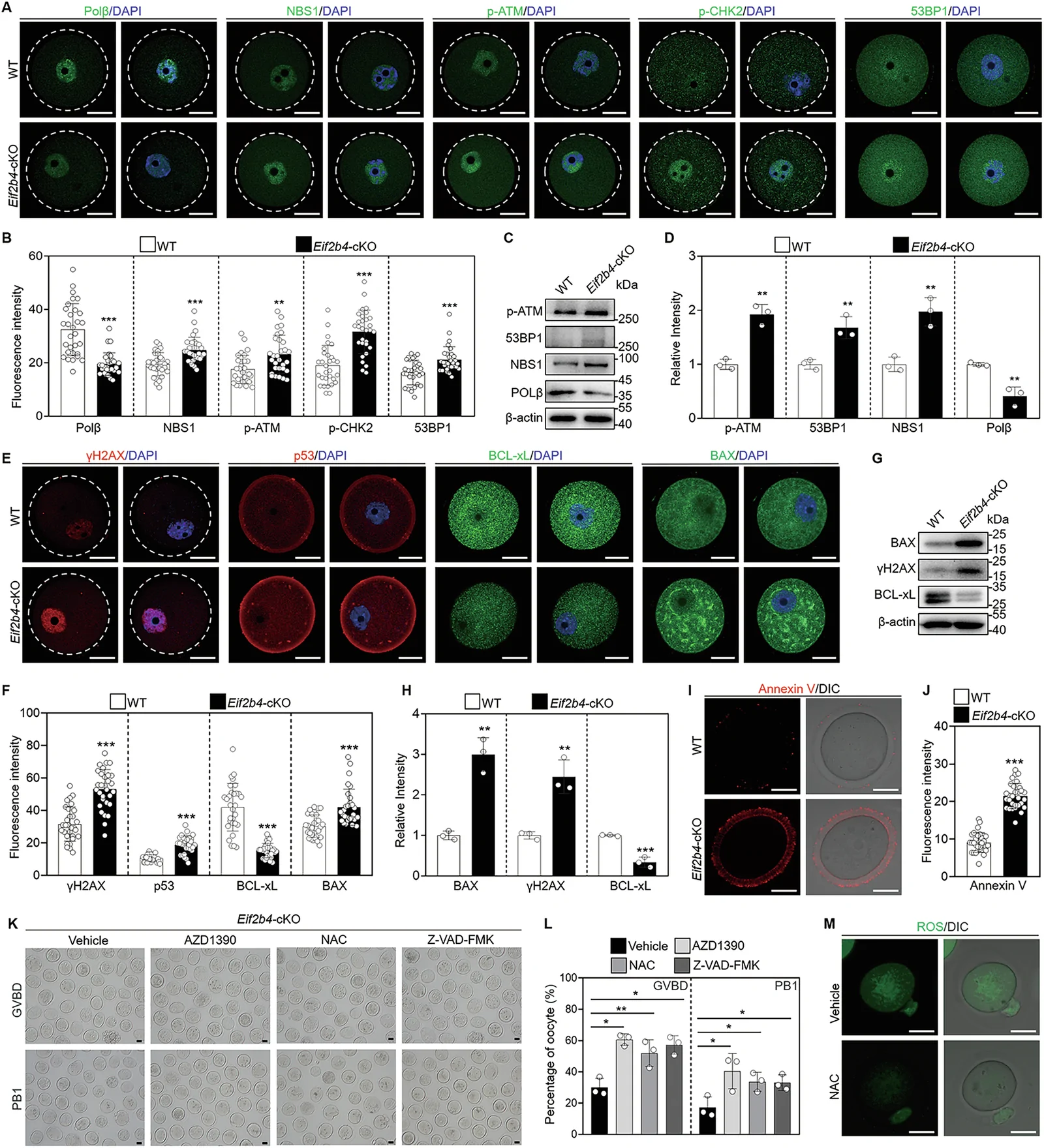
*Eif2b4* deletion in oocytes induces DNA damage and oocyte apoptosis. **A** Immunofluorescence staining of Polβ, NBS1, p-ATM, p-CHK2, and 53BP1 in WT and cKO oocytes. **B** Quantification of fluorescence intensity of Polβ, NBS1, p-ATM, p-CHK2, and 53BP1 (*n* = 30 oocytes). **C**, **D** Western blot analysis of Polβ, NBS1, p-ATM, and 53BP1 expression in WT and cKO oocytes. **E** Immunofluorescence staining of γH2AX, p53, BAX, and BCL-xL in WT and cKO oocytes. **F** Quantification of fluorescence intensity of γH2AX, p53, BAX, and BCL-xL (*n* = 30 oocytes). **G**, **H** Western blot analysis of γH2AX, BAX, and BCL-xL expression in WT and cKO oocytes. **I** Annexin-V fluorescent staining in WT and cKO oocytes. **J** Quantification of fluorescence intensity of Annexin-V (*n* = 30 oocytes). **K** Representative images of GVBD and PB1 extrusion in *Eif2b4*-cKO oocytes after 14 h culture with vehicle (0.1% DMSO), AZD1390 (3 nM), NAC (2 mM), or Z-VAD-FMK (20 μM). **L** Quantification of GVBD and PB1 rates. **M** Representative images of ROS levels in *Eif2b4*-cKO oocytes treated with vehicle or NAC. In each experiment, *n* ≥ 3 biological replicates. Bars indicate the mean ± SD. A two-sided Student’s *t* test was used to determine *P* values. (**P < 0.01 and ***P < 0.001). Scale bar: 25 μm.

Excessive accumulated ROS and decreased DNA repair capacity both trigger DNA damage, which then activates the apoptotic pathway in tubular cells [38]. Immunofluorescence staining and Western blot analysis showed significant alterations in the expression levels of DNA damage repair-related proteins in *Eif2b4*-cKO oocytes compared with those of WT oocytes. Specifically, both the fluorescence intensity and protein levels of DNA polymerase beta (Polβ) were significantly decreased, while those of NBS1, phosphorylated ataxia-telangiectasia mutated (p-ATM), and p53-binding protein 1 (53BP1) increased substantially (Fig. 7A–D). Correspondingly, the fluorescence intensity and protein levels of the DNA damage marker phosphorylated H2AX (γH2AX) were also significantly increased in *Eif2b4*-cKO oocytes compared with those of WT oocytes (Fig. 7E–H). Furthermore, a significant increase in the fluorescence intensity of DDR mediators, phosphorylated CHK2 (p-CHK2), and p53 was detected in *Eif2b4*-cKO oocytes (Fig. 7A, B, E, F). As expected, *Eif2b4*-cKO oocytes exhibited increased fluorescence intensity and protein levels of the pro‑apoptotic protein BAX, alongside reduced fluorescence intensity and protein levels of the anti‑apoptotic protein BCL‑xL (Fig. 7E–H). A significantly increased fluorescence intensity of Annexin-V was detected in *Eif2b4*-cKO oocytes (Fig. 7I, J), indicating oocyte apoptosis. Moreover, NAC (an antioxidant) treatment significantly decreased ROS levels and Annexin-V fluorescence intensity, and increased the rates of GVBD and PB1 in *Eif2b4*-cKO oocytes (Fig. 7K–M and Supplementary Fig. 6A–C). In addition, short-term treatment with AZD1390 (an ATM inhibitor) or Z-VAD-FMK (a pan-caspase inhibitor) significantly decreased the apoptosis rate of *Eif2b4*-cKO oocytes and increased the percentage of oocytes that undergo GVBD and PB1 (Fig. 7K, L and Supplementary Fig. 6B, C). The findings suggest that the depletion of *Eif2b4* in oocytes triggers DNA damage-induced apoptotic pathways, ultimately leading to oocyte apoptosis.

## Discussion

**Fig. 8:**
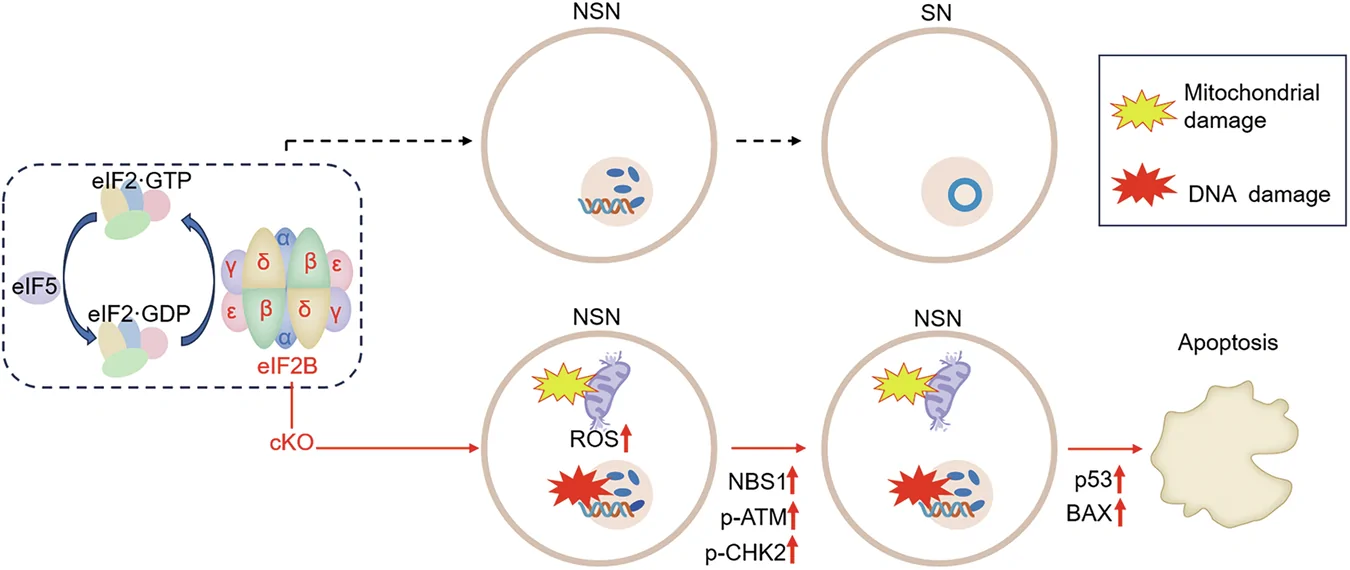
Schematic diagram of eIF2B subunits involved in translation initiation and oocyte growth. Oocyte growth requires massive protein synthesis. eIF2B plays a crucial role in translation initiation by mediating the guanine nucleotide exchange on eIF2. Deletion of *Eif2b2*, *Eif2b3*, *Eif2b4*, or *Eif2b5* in mouse oocytes impairs mitochondrial function and reduces DNA damage repair capacity. Subsequently, the persistent DNA damage activates the DNA damage response pathway and apoptotic pathway, leading to oocyte apoptosis within early follicles.

Clinically, mutations in the *EIF2B* lead to ovarioleukodystrophy, characterized by the co-occurrence of leukodystrophy and POI [39]. Our study demonstrated that oocyte-specific knockout of *Eif2b2, Eif2b3, Eif2b4*, or *Eif2b5* resulted in oocyte apoptosis within early follicles. The underlying mechanism involved DNA damage-induced DDR, impaired mitochondrial function, and activation of the apoptotic pathway (Fig. 8).

Histomorphological analysis revealed the presence of secondary follicles, and antral follicles were observed in *Eif2b* cKO mice, suggesting that the follicles with eIF2B deletion can still develop into advanced stages. This observed phenotype may be explained by the following possible reasons. First, functional compensation may exist between different eIF2B subunits. Second, the residual eIF2B subunits from oocytes within primordial follicles (as shown in immunofluorescence staining and Western blot data) maintain follicle development in the cKO mice. Third, the reduced ubiquitination levels (as shown in Ribo-seq and RNA-seq data) are also beneficial for slowing down the degradation of residual eIF2B subunits in cKO mice. Finally, both Ribo-seq and RNA-seq showed upregulation of numerous ISR-related genes at both the mRNA and translation levels, demonstrating activation of ISR in *Eif2b4*-cKO oocytes. Consistent with reports in other cells [40, 41], the moderate activation of ISR also helps in maintaining oocyte growth and follicle development in cKO mice by facilitating critical protein translation.

Interestingly, among the five subunits of eIF2B, deletion of *Eif2b1* had no effect on follicle development, whereas the deletion of *Eif2b4* resulted in the most severe defects. This difference may be attributed to the fact that deletion of *Eif2b1* in oocytes had almost no impact on protein translation, while deletion of *Eif2b4* caused a pronounced reduction in global translation. These results indicate that deficiency in different eIF2B subunits shows varying degrees of impact on protein translation. A potential mechanism underlying the severe defects caused by *Eif2b4* deletion is that it serves as a critical scaffold within the eIF2B complex. The loss of eIF2Bδ not only completely disrupts decamer assembly but also reduces TC availability, thereby leading to a more profound translation impairment [42, 43]. Furthermore, hydrophobic residues within the eIF2Bδ subunit of the intact eIF2B complex mediate the interaction with p-eIF2α, thereby sensing eIF2α phosphorylation [15]. This leads to decreased p-eIF2α levels and disruption of translational homeostasis [42].

Deletion of *Eif2b4* in oocytes resulted in the downregulation of DNA damage repair protein Polβ and anti-apoptotic protein BCL-xL, as well as the upregulation of DDR proteins (NBS1, p-ATM, p-CHK2, and p53) and proapoptotic protein BAX. Furthermore, the *Eif2b4* deletion also reduced mitochondrial membrane potential, mtDNA copy numbers, and ATP levels, and triggered an accumulation of ROS, suggesting mitochondrial dysfunction [44–46]. The downregulation or mutation of Polβ leads to significant accumulation of DNA damage in breast and ovarian cancer cells [47, 48], and excessive ROS can also cause DNA damage in retinal precursor cell line R28 [49], GC-1 [50], and breast cancer cells [51]. ATM is activated at DNA damage sites through its interaction with the MRE11-RAD50-NBS1 (MRN) complex, leading to the phosphorylation of DDR proteins, including H2AX, MRN complex, and CHK2 [52–54]. When DNA damage is not rapidly rectified, the CHK2-p53-BAX pathway is activated, resulting in cell apoptosis [55, 56]. Therefore, the deletion of *Eif2b4* leads to DNA damage and ROS, and then activates the DDR and apoptotic pathways, leading to the apoptosis of oocytes at the early follicular stage. *Eif2b2*-, *Eif2b3*-, and *Eif2b5*-cKO mice showed similar phenotypes but exhibited different degrees of follicle development disorders. Future work should be done to indicate whether eIF2B2, eIF2B3, or eIF2B5 shares a similar regulatory mechanism in follicle development as that of eIF2B4.

The depletion of *Eif4e1b* in primary oocytes leads to oocyte development defects and early embryonic developmental arrest by downregulating maternal mRNA translation [27, 57]. Depleting the translation regulatory factor *Zar1/2* in germ cells leads to defects in oocyte meiosis maturation by impairing polyadenylation and zygotic genome activation [58]. The depletion of *Eif2b* subunits, *Eif5*, and *Eif2s2* in oocytes, as well as *Eif2s2* in premeiotic germ cells, both resulted in oocyte apoptosis mediated by DNA damage and ROS accumulation [28, 29, 59]. The possible reason for the phenotypic difference is that the depletion of *Eif2b* subunits*, Eif2s2* and *Eif5* downregulates the global protein translation, whereas the depletion of *Zar1/2* and *Eif4e1b* specifically impairs the selective mRNA translation in oocytes.

Clinical studies have shown that mutations in the *EIF2B* genes can lead to leukodystrophy that is often accompanied by POI, but the specific pathogenic mechanisms have not yet been systematically elucidated. In the present study, the deletion of *Eif2b4* resulted in apoptosis in oocytes and granulosa cells, which was consistent with the recently reported phenotype of granulosa cell apoptosis caused by *EIF2B5* mutations in humans [60]. Mechanistically, both *Eif2b4* deletion in mouse oocytes and *EIF2B5*-mutant human granulosa cells exhibited oxidative stress and ISR activation. Interestingly, the treatment with NAC (an antioxidant) can increase the oocyte maturation rate in *Eif2b4*-cKO mice in the present study, and has also been reported to alleviate *EIF2B5* mutation-induced granulosa cell apoptosis [60]. Given the efficacy of ISRIB in treating cardiovascular, oncological, and neurodegenerative disorders, as well as ovarian dysfunction [61, 62], targeting the ISR may be a potential therapeutic strategy to rescue follicle development defects driven by EIF2B dysfunction. Although DNA damage and DDR pathway activation were not detected in human granulosa cells with *EIF2B5* mutations [60], transient treatment with an ATM inhibitor reduced oocyte apoptosis in the *Eif2b4* deletion mice, suggesting that inhibitors of DNA damage or the DDR pathway may be used to treat patients with severe EIF2B mutations

In conclusion, our study demonstrates that the deletion of *Eif2b* subunits leads to the arrest of early growing follicle development and oocyte apoptosis. The underlying mechanisms involve DNA damage-induced DDR, impaired mitochondrial function, and activation of the apoptotic pathway. The mechanism of this study provides valuable insights into the genetic diagnosis and clinical treatment of POI.

## Supplementary information

Supplementary figures and tables

Original data

## Data Availability

The Ribo-seq and RNA-seq raw sequencing data generated in this study have been deposited in the NCBI Sequence Read Archive (SRA) database under accession numbers PRJNA1416251 and PRJNA1417315, respectively. All data supporting the findings of this study are available within the article and/or the supplementary information. Additional data related to this paper may be requested from the authors.
