# Supplementary material for: Oocyte-specific knockout of eIF2B subunits causes apoptosis of mouse oocytes within the early growing follicles via DNA damage and mitochondrial dysfunction
Source: Cell Death Dis. 2026 Jul 10;17(1):858. doi: 10.1038/s41419-026-09092-3 (PMC13639025; doi:10.1038/s41419-026-09092-3)

Fig. 1D

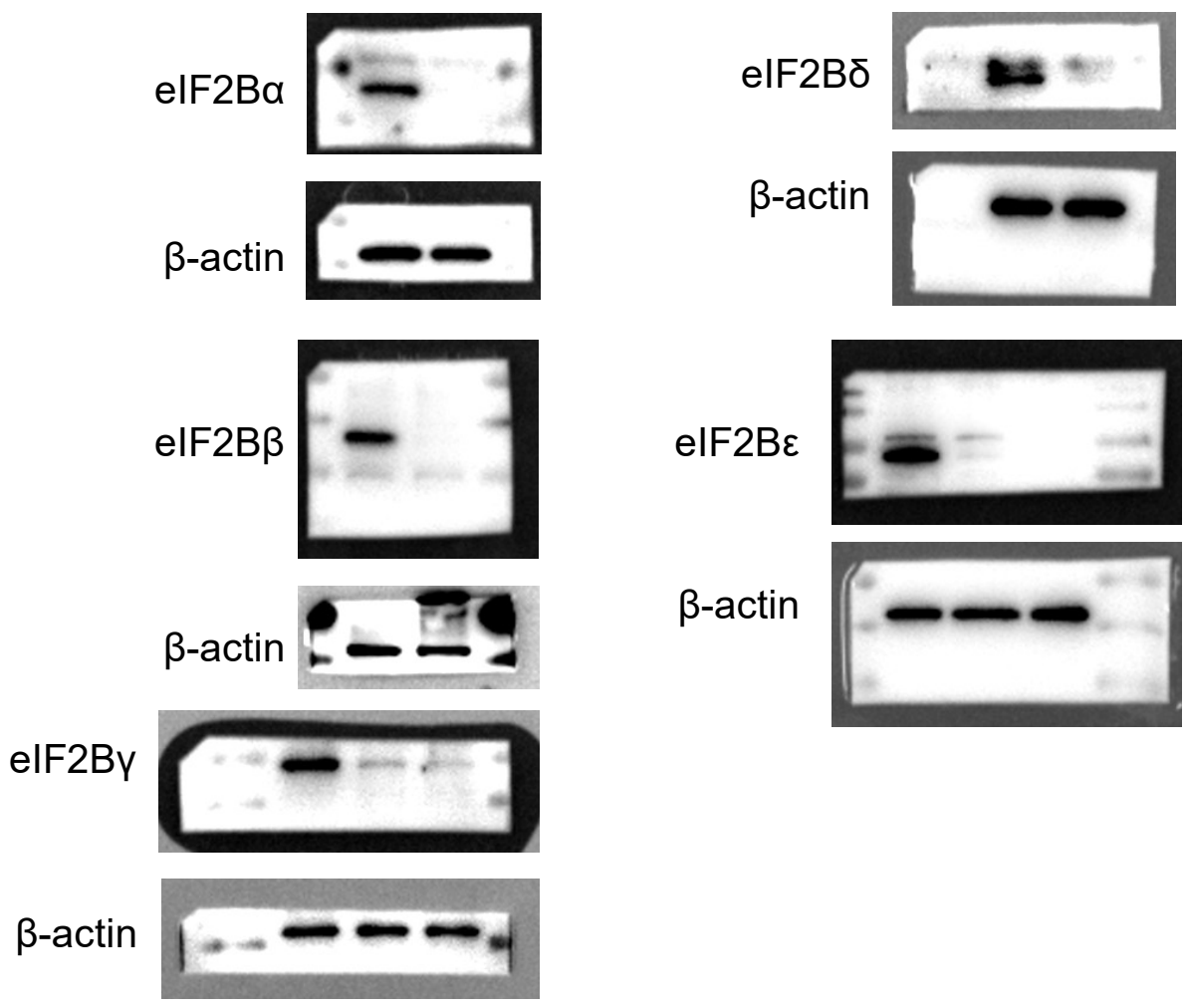

Fig. 3C

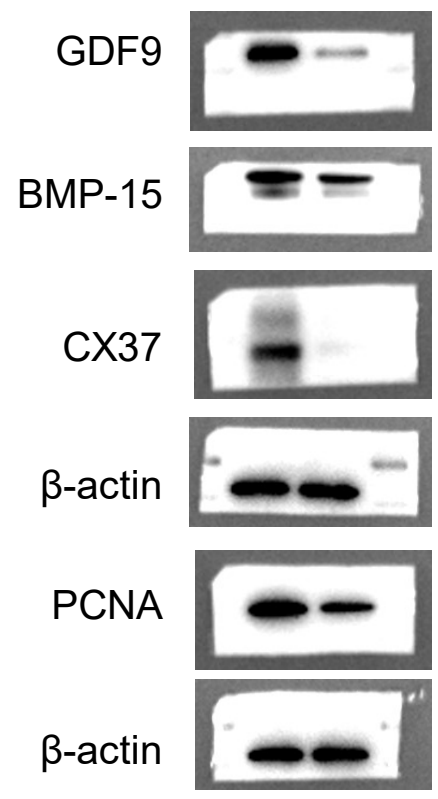

Fig. 4A

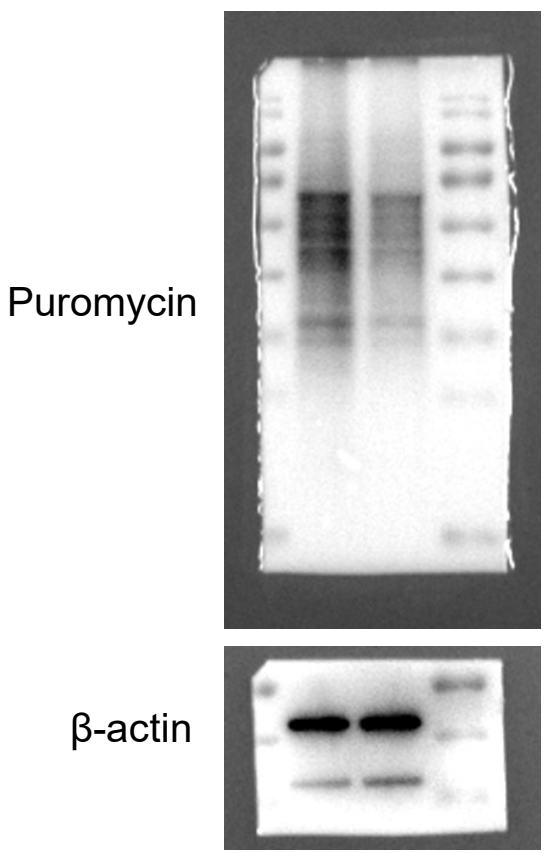

Fig. 4F

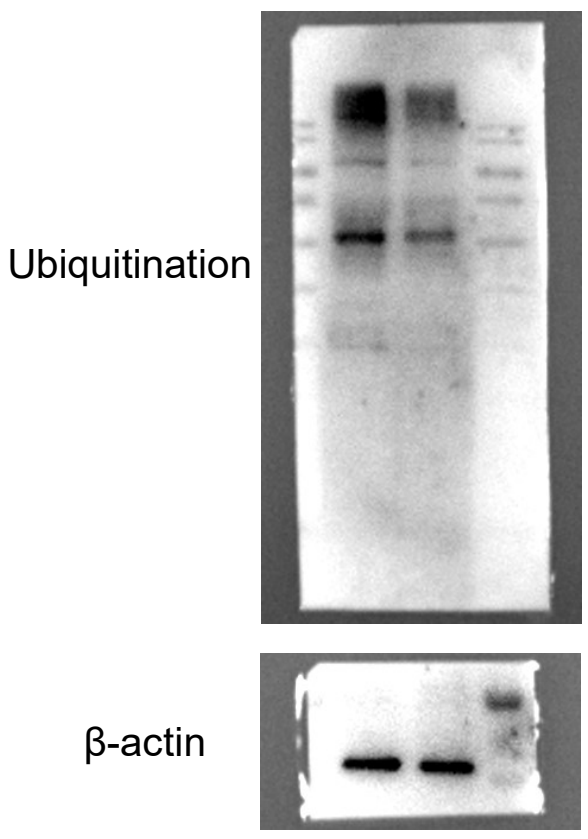

Fig. 6C

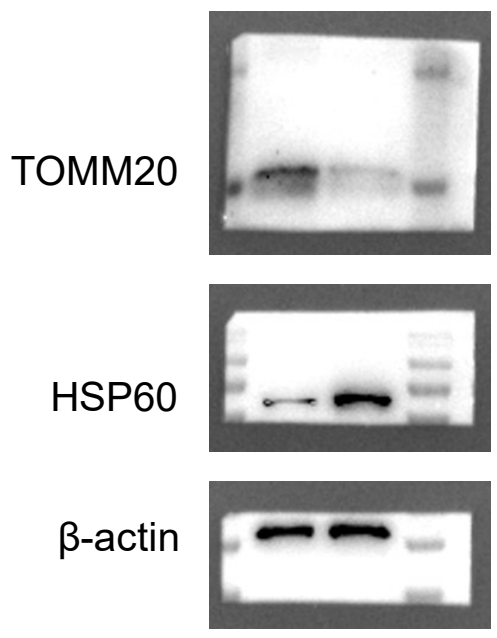

Fig. 7C

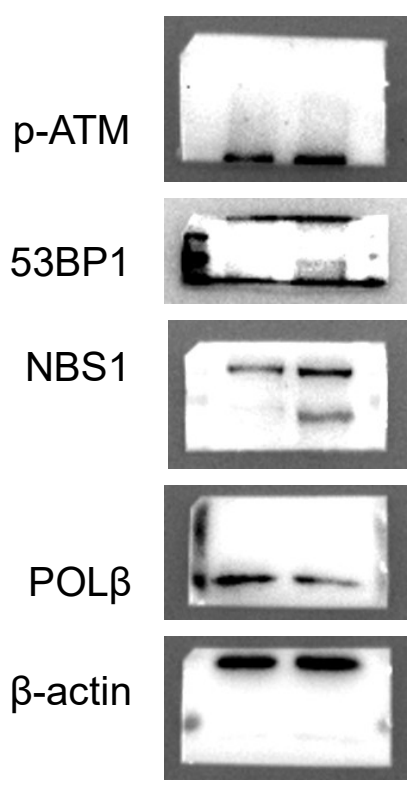

Fig. 7G

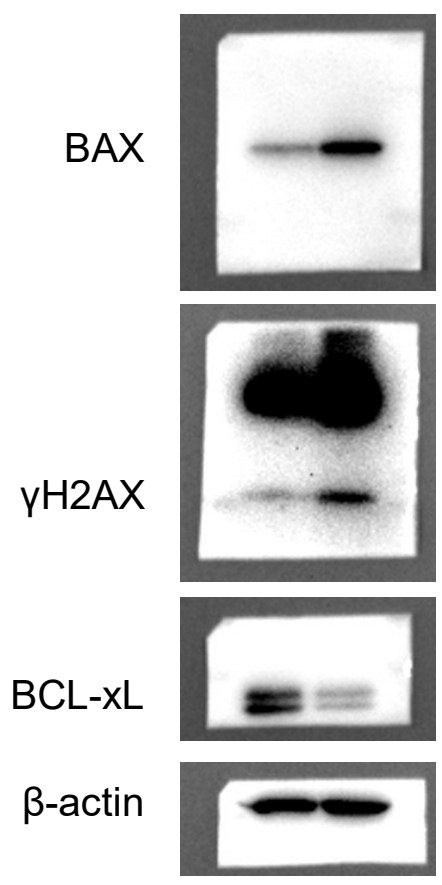

Supplementary Fig. 4A

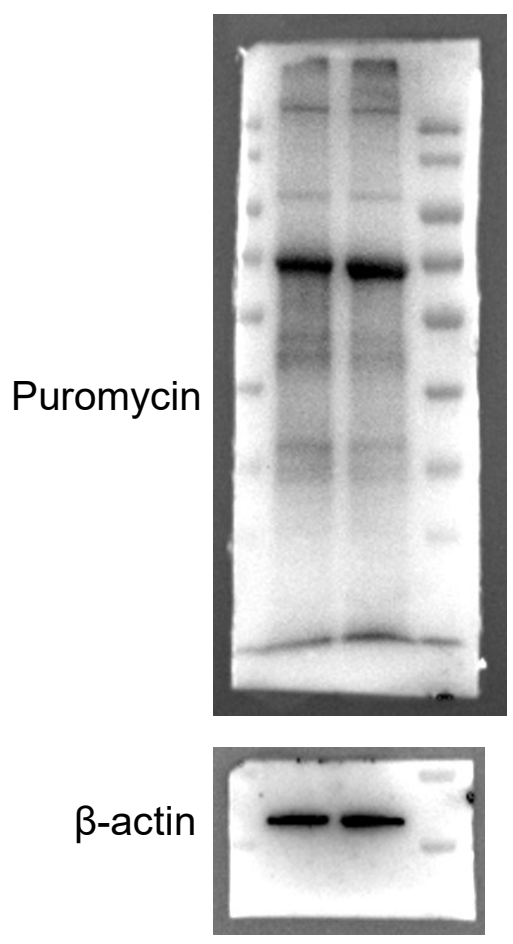

Supplement: Supplementary file 1 [file 41419_2026_9092_MOESM2_ESM.pdf]
