# Supplementary material for: Oocyte-specific knockout of eIF2B subunits causes apoptosis of mouse oocytes within the early growing follicles via DNA damage and mitochondrial dysfunction
Source: Cell Death Dis. 2026 Jul 10;17(1):858. doi: 10.1038/s41419-026-09092-3 (PMC13639025; doi:10.1038/s41419-026-09092-3)
Supplement: Supplementary file 2 [file 41419_2026_9092_MOESM1_ESM.docx]

**Supplementary figures and tables**

**Supplementary Fig. 1 *Eif2b* subunits deletion in oocytes impairs follicle development. A** Schematic representation of *Eif2b* subunits deletion by *Gdf9*-Cre-mediated recombinase in oocytes. **B** Representative images of ovaries isolated from WT and cKO female mice at the indicated age. **C** Quantiﬁcation of the number of follicles at different developmental stages, n = 3 females for each genotype. Bars indicate the mean ± SD. A two-sided Student’s t-test was used to determine *P* values. (****P* < 0.001). Scale bar: 100 μm.

**Supplementary Fig. 2 *Eif2b4* deletion in oocytes impairs histone methylation in oocytes.** **A, B** Immunofluorescence staining of H3K4me3 and H3K27me3 in WT and cKO oocytes. Scale bars: 25 μm.

**Supplementary Fig. 3 *Eif2b4* deletion in oocytes impairs the bidirectional communication between the oocyte and granulosa cells. A** Immunofluorescence staining of CX37, ZP1 and ZP3 in WT and cKO ovaries at PD21. **B** Immunofluorescence staining of Ki-67, PCNA and CX43 in WT and cKO ovaries at PD21. **C** Immunofluorescence staining of BrdU, TUNEL and c-CASP-3 (cleaved Caspase-3) in WT and cKO ovaries at PD21. **D** Representative images of PCNA immunofluorescence staining in granulosa cells after co-culture with WT or *Eif2b4*-cKO oocytes for 48 h. **E** Quantification of the percentage of PCNA-positive granulosa cells. In each experiment, n ≥ 3 biological replicates. Bars indicate the mean ± SD. A two-sided Student’s t-test was used to determine *P* values. (****P* < 0.001). Scale bars: 100 μm (A, B, C, D).

**Supplementary Fig. 4 The integration analysis between transcriptomic and translatome in *Eif2b4*-cKO oocytes.** **A, B** Western blot analysis of puromycin incorporation in WT and *Eif2b1*-cKO oocytes. **C** Distribution of the read lengths for Ribo-seq. **D** Distribution of Ribo-seq coverage in different gene regions. **E** Volcano plot of differentially translated efficiency genes (DTEGs) between WT and cKO oocytes. **F** Nine-quadrant analysis of transcriptome and translation efficiency in WT and cKO oocytes. Dashed lines indicate thresholds for transcriptome (FC ≥ 2.0) and translation efficiency (FC ≥ 1.5). In each experiment, n ≥ 3 biological replicates. Bars indicate the mean ± SD.

**Supplementary Fig. 5 *Eif2b5* deletion in oocytes impairs the integrity of the transcriptome. A** Volcano plot illustrating the differentially expressed transcripts in WT and cKO oocytes. **B, C** Bubble chart illustrating the enriched GO terms associated with the significantly downregulated and upregulated transcripts in cKO oocytes. In each experiment, n ≥ 3 biological replicates.

**Supplementary Fig. 6 NAC, AZD1390, and Z-VAD-FMK reduce apoptosis in *Eif2b4*-cKO oocytes. A** Quantification of relative ROS fluorescence intensity (n = 30 oocytes). **B** Representative images of Annexin V staining. **C** Quantiﬁcation of fluorescence intensity of Annexin-V (n = 30 oocytes). In each experiment, n ≥ 3 biological replicates. Bars indicate the mean ± SD. A two-sided Student’s t-test was used to determine *P* values. (***P* < 0.01). Scale bar: 25 μm.

**Table S1. Primers for PCR**

| Primer name | Genes targeted | Application | Sequences (5’-3’) |
| --- | --- | --- | --- |
| FRT-F | *Eif2b1* | Identification of *Eif2b1* flox | TTTCGTTCTCATGGGATGGCC |
| FRT-R |  |  | AGTAGGCAAACTGCTTTGTGGTGC |
| FRT-F | *Eif2b2* | Identification of *Eif2b2* flox | CAGGCACACATGAGGCAAACAG |
| FRT-R |  |  | AGGCTCAGAGTGCAACAGTCCAAG |
| FRT-F | *Eif2b3* | Identification of *Eif2b3* flox | ATATCACCAAAGCTCAGCCCAGC |
| FRT-R |  |  | GATTCACTAAGCCTCTCCTTGCCA |
| FRT-F | *Eif2b4* | Identification of *Eif2b4* flox | GCAGACCAAGAAATTGGCTCTGC |
| FRT-R |  |  | CCAACTGACCTTGGGCAAGAACAT |
| FRT-F | *Eif2b5* | Identification of *Eif2b5* flox | TTTAACCTTGCAGGACATGGGC |
| FRT-R |  |  | AACTATGGATTGCTGAGCGACC |
| Cre-R1 | *Gdf9*-Cre | Identification of *Gdf9*-Cre | AAGAACCTGATGGACATGTTCAG |
| Cre-R2 |  |  | CTGATTCTGGCAATTTCGG |

**Table S2. Primers for qRT-PCR**

| Genes | Forward primers (5’-3’) | Reverse primers (5’-3’) |
| --- | --- | --- |
| *Asns* | GCAGTGTCTGAGTGCGATGAA | TCTTATCGGCTGCATTCCAAAC |
| *Atf3* | CCCCTGGAGATGTCAGTCAC | CGGTGCAGGTTGAGCATGT |
| *Atf4* | CCTATAAAGGCTTGCGGCCA | CACTGCTGCTGGATTTCGTG |
| *Chac1* | CTGTGGATTTTCGGGTACGG | CCCCTATGGAAGGTGTCTCC |
| *Ddit3* | CTGGAAGCCTGGTATGAGGAT | CAGGGTCAAGAGTAGTGAAGGT |
| *Eif4e1b* | GGGGACTACAGCACCACTC | CTCATCGCTGGTAGGGCTA |
| *Fgf21* | CTGCTGGGGGTCTACCAAG | CTGCGCCTACCACTGTTCC |
| *Herpud1* | GCAGTTGGAGTGTGAGTCG | TCTGTGGATTCAGCACCCTTT |
| *Hmox1* | AGGTACACATCCAAGCCGAGA | CATCACAGCTTAAAGCCTTCT |
| *Inhbe* | CTAACCAGCCGTCCCAGAATA | GTGCCCGGAAAAGAGGGAG |
| *Mrps26* | CATGACCCGCCTGCCAAAT | TCTCCCGGTACTGTCGGTAG |
| *Slc7a3* | TGAGCACCCTCGACTTAGTG | CACAATGGATGGTCCTGCTTTA |
| *Trib3* | ACCTTCAGAGCGACTTGTGGG | GCTTGGCCCAAAAAGTCAGG |
| *Xbp1* | CTGAGTCCGCAGCAGGTG | TTCCAGCTTGGCTGATGAGG |
| *Rpl19* | CTGAAGGTCAAAGGGAATGTGTTC | TGGTCAGCCAGGAGCTTCTTG |

**Table S3. List of primary antibodies used in immune detection**

| Antibody | Catalog Code | | Source | | Host | | | Dilution | |
| --- | --- | --- | --- | --- | --- | --- | --- | --- | --- |
|  |  |  |  |  |  |  |  | IF | WB |
| 53BP1 | | A3859 | | Abclonal | | Rabbit | 1:200 | | 1:1000 |
| BMP15 | | 18982-1-AP | | Proteintech | | Rabbit | 1:100 | | 1:1000 |
| BAX | | 50599-2-Ig | | Proteintech | | Rabbit | 1:200 | | 1:1000 |
| BCL-xL | | 2764 | | CST | | Rabbit | 1:200 | | 1:1500 |
| BrdU | | ab1893 | | Abcam | | Sheep | 1:200 | | — |
| CX37 | | CX37A11-A | | Alpha Diagnostic International | | Mouse | 1:100 | | 1:1000 |
| CX43 | | 26980-1-AP | | Proteintech | | Rabbit | — | | 1:1000 |
| Cleaved Caspase-3 | | 9664 | | CST | | Rabbit | 1:50 | | — |
| eIF2B1 | | A7892 | | Abclonal | | Rabbit | 1:200 | | 1:1000 |
| eIF2B2 | | A7027 | | Abclonal | | Rabbit | 1:200 | | 1:1000 |
| eIF2B3 | | A10262 | | Abclonal | | Rabbit | 1:200 | | 1:1000 |
| eIF2B4 | | A18203 | | Abclonal | | Rabbit | 1:200 | | 1:1000 |
| eIF2B5 | | A8670 | | Abclonal | | Rabbit | 1:200 | | 1:1000 |
| GDF9 | | ab254323 | | Abcam | | Rabbit | 1:200 | | 1:1000 |
| H3K4me3 | | ab8580 | | Abcam | | Rabbit | 1:200 | | — |
| H3K27me3 | | P37961-3F | | Abmart | | Rabbit | 1:200 | | — |
| HSP60 | | 46611S | | CST | | Rabbit | — | | 1:1000 |
| Ki-67 | | 9129s | | CST | | Rabbit | 1:200 | | — |
| NBS1 | | A4197 | | Abclonal | | Rabbit | 1:200 | | 1:1000 |
| p53 | | sc-126 | | Santa Cruz | | Rabbit | 1:100 | | — |
| PCNA | | 2586 | | CST | | Mouse | 1:200 | | 1:1000 |
| Polβ | | A1681 | | Abclonal | | Rabbit | 1:200 | | 1:1000 |
| Puromycin | | A23031 | | Abclonal | | Rabbit | — | | 1:1000 |
| Phospho-ATM-S1981 | | AP0008 | | Abclonal | | Rabbit | 1:200 | | 1:1000 |
| Phospho-CHK2-T68 | | HY-80799 | | MCE | | Rabbit | 1:200 | | — |
| TOMM20 | | A19403 | | Abclonal | | Rabbit | 1:200 | | 1:1000 |
| Ubiquitin | | PTM-5798 | | PTM | | Rabbit | — | | 1:500 |
| ZP1 | | sc-32751 | | Santa Cruz | | Mouse | 1:100 | | — |
| ZP3 | | sc-398359 | | Santa Cruz | | Mouse | 1:100 | | — |
| α-tubulin | | ab195887 | | Abcam | | — | 1:400 | | — |
| γH2AX | | ab206900 | | Abcam | | Mouse | — | | 1:500 |
| γH2AX | | ab22551 | | Abcam | | — | 1:300 | | — |
| β-actin | | 4967 | | CST | | Rabbit | — | | 1:1000 |

IF: Immunofluorescence; WB: Western blotting

**Table S4. Primers for quantification of mtDNA copy number**

| Genes | Forward primers (5’-3’) | Reverse primers (5’-3’) |
| --- | --- | --- |
| *ND1* | CTAGCAGAAACAAACCGGGC | CCGGCTGCGTATTCTACGTT |
| *HK2* | GCCAGCCTCTCCTGATTTTAGTGT | GGGAACACAAAAGACCTCTTCTGG |
